# Five biopsy specimens from the proximal part of the tumor reliably determine HER2 protein expression status in gastric cancer

**DOI:** 10.1007/s10120-015-0502-3

**Published:** 2015-05-19

**Authors:** Naoyuki Tominaga, Takuji Gotoda, Megumi Hara, Matthew D. Hale, Takayoshi Tsuchiya, Jun Matsubayashi, Shin Kono, Chika Kusano, Takao Itoi, Kazuma Fujimoto, Fuminori Moriyasu, Heike I. Grabsch

**Affiliations:** Department of Gastroenterology and Hepatology, Tokyo Medical University, Tokyo, Japan; Departments of Internal Medicine and Gastrointestinal Endoscopy, Saga Medical School, Saga, Japan; Department of Preventive Medicine, Faculty of Medicine, Saga Medical School, Saga, Japan; Section of Pathology and Tumour Biology, Leeds Institute of Cancer and Pathology, University of Leeds, Leeds, UK; Department of Pathology, Tokyo Medical University, Tokyo, Japan; Department of Pathology, Maastricht University Medical Center, P. Debyelaan 25, 6202 AZ Maastricht, The Netherlands; GROW School for Oncology and Developmental Biology, Maastricht University Medical Center, Maastricht, The Netherlands

**Keywords:** HER2 expression, Gastric cancer, Virtual biopsy

## Abstract

**Background:**

National guidelines recommend trastuzumab for treatment of patients with metastatic HER2-positive gastric cancer (GC). There is currently no guideline indicating the number of biopsy specimens and the location from which they should be obtained to reliably determine the human epidermal growth factor receptor 2 (HER2) status in GC. The aim of this pilot study was (a) to quantify HER2-positive tumor cells in different tumor regions to assess the spatial heterogeneity of HER2 expression and (b) to establish the required number of biopsy specimens and the location from which they should be obtained within the tumor to achieve concordance between HER2 expression status in the biopsy specimens and the resection specimen.

**Methods:**

HER2 expression was quantified in six different regions of 24 HER2-positive GC and in six virtual biopsy specimens from different luminal regions. Intratumoral regional heterogeneity and concordance between HER2 status in the biopsy specimens and the resection specimen were analyzed.

**Results:**

HER2-positive cells were more frequent in the luminal tumor surface compared with deeper layers (*p* < 0.001). GCs with differentiated histological features were more commonly HER2 positive (*p* < 0.001). Assessment of HER2 expression status in five biopsy specimens was sufficient to achieve 100 % concordance between the biopsy specimens and the resection specimen.

**Conclusions:**

This is the first study to suggest preferential HER2 positivity at the luminal surface in GC and to establish a minimum number of biopsy specimens needed to obtain a biopsy HER2 result which is identical to that from the whole tumor. Our study suggests that HER2 testing in five tumor-containing endoscopic biopsy specimens from the proximal (oral) part of the tumor is advisable. The results from this pilot study require validation in a prospective study.

## Introduction

Gastric cancer (GC) incidence has been steadily declining in recent decades, but GC remains the fifth commonest cancer globally and the second commonest cancer in Japan [1]. GC patients with inoperable, metastatic or recurrent disease have very poor survival even after palliative cytotoxic chemotherapy [2]. The ToGA trial demonstrated that trastuzumab therapy in combination with cytotoxic chemotherapy significantly improved survival of patients with human epidermal growth factor receptor 2 (HER2)-positive GC [3]. Since then, trastuzumab has been licensed in Europe [4] and the USA and other countries for use in HER2-positive metastatic GC [5].

With the exception of patients with recurrent disease after previous resection, the HER2 status is usually determined using endoscopic biopsy specimens. GC-specific HER2 scoring guidelines have been established recently, detailing that a GC biopsy specimen with complete, basolateral or lateral membranous immunoreactivity in more than five “clustered tumor cells” is classified as HER2 positive [6]. However, the same HER2 guidelines are vague regarding the number of biopsy specimens, the tumor content per biopsy specimen, or the location from which the biopsy specimen should be obtained within the tumor, and simply recommend that “an adequate number of viable biopsy specimens (ideally six to eight) are required.” The current guidelines do not provide any evidence for the recommendations made and do not seem to consider the suggested relationship between HER2 positivity and histological subtype, grade of differentiation [7–11], and tumor location [12] in GC.

The reported concordance of GC HER2 expression between endoscopic biopsy specimens and resection material ranges widely from 45.5 to 88.5 % [13, 14]. Investigators have mostly provided only very little or no information at all on the resection and/or biopsy material used, and have concluded that this variability is due to “intratumoral heterogeneity” [15] without providing a definition of this term.

We hypothesized (a) that HER2-positive tumor cells have no preferential spatial distribution within a given GC and (b) that a high concordance of the HER2 status between biopsy specimens and the resection specimen can be achieved with six tumor-containing biopsy specimens. We decided on six biopsy specimens on the basis of the ideal number of biopsy specimens mentioned above.

To test the above-mentioned hypotheses, we (a) quantified HER2-positive tumor cells in six different regions in gastric resection specimens to measure the spatial HER2 expression heterogeneity and (b) assessed HER2 expression status in six virtual biopsy specimens from different luminal locations within the tumor to calculate the concordance rate between biopsy specimen and resection specimen HER2 status.

## Materials and methods

Eighty-four patients were treated by gastrectomy for gastric adenocarcinoma at the Tokyo Medical University between 2011 and 2013. In all cases, the tumor was sampled from anal to oral, including the deepest tumor infiltration, at the time of cutting up the specimen according to our routine laboratory protocol [16].

HER2 expression was investigated in tumor blocks per case using immunohistochemistry (HercepTest II, Dako, Japan) according to the instructions of the manufacturer and using previously published scoring criteria [17]. The tumor slice with the deepest tumor invasion was used for this study. Depending on the tumor size, this slice was embedded in one or more cassettes. The maximum number of cassettes required to completely embed the tumor slices with the deepest invasion was six.

Twenty-four GCs were classified as HER2 positive on the basis of immunohistochemistry (HER2 scores of 2+ and 3+) and were included in the current study. *HER2* copy number was not assessed.

The macroscopic tumor type was classified according to the Japanese classification of GC [16]. The depth of invasion (T category) and the lymph node status (N category) were classified using the seventh edition of the Union for International Cancer Control TNM classification [18]. The histological tumor type was classified using the Japanese classification and the Lauren classification [16, 18].

**Fig. 1:**
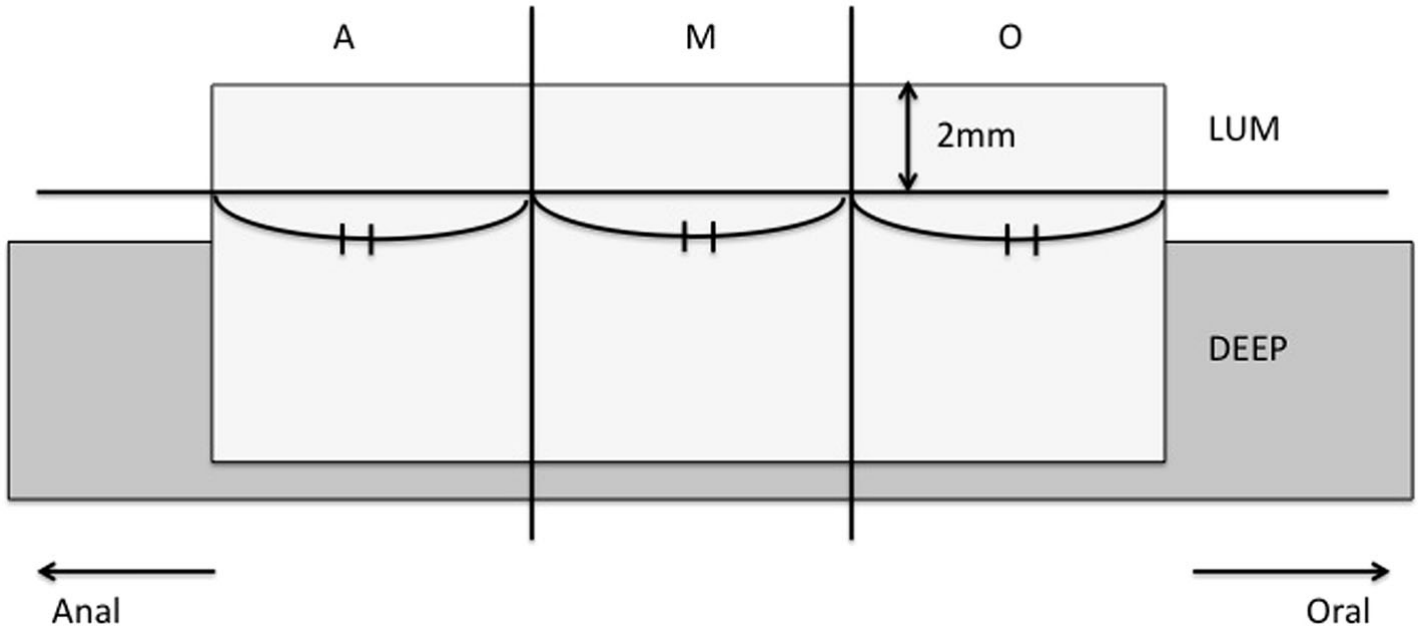
The division of the tumor into six parts. The tumor was divided equally into three regions—oral region (*O*), central region (*M*), and anal region (*A*)—which were each divided in turn into an upper layer (*LUM*) and a lower layer (*DEEP*)

For the assessment of the spatial heterogeneity of HER2 expression in the tumor, every tumor was divided into three regions of equal length designated as “oral” [i.e., from the oral (proximal) tumor edge to the central part of the tumor], “central” (i.e., the central part of the tumor), and “anal” [i.e., from the central part of the tumor to the anal (distal) tumor edge]; see Fig. 1. The median length of the each individual region was 14.7 mm, ranging from 5 to 40 mm. Each tumor region was further divided into a luminal layer measuring 2 mm from the luminal tumor surface into the wall and a deeper layer comprising all tumor beyond the 2-mm depth limit; see Fig. 1. A 2-mm cutoff was chosen as this is the depth that can be sampled by routine endoscopic biopsy (disposable biopsy forceps, Olympus, Japan). The histological tumor type was determined for each region separately. The percentage of HER2-positive tumor cells was established for each region by assessing 200 tumor cells in ten randomly selected areas per region—that is, by assessing a total of 2000 tumor cells per region and up to a total of 12,000 tumor cells per case.

**Fig. 2:**
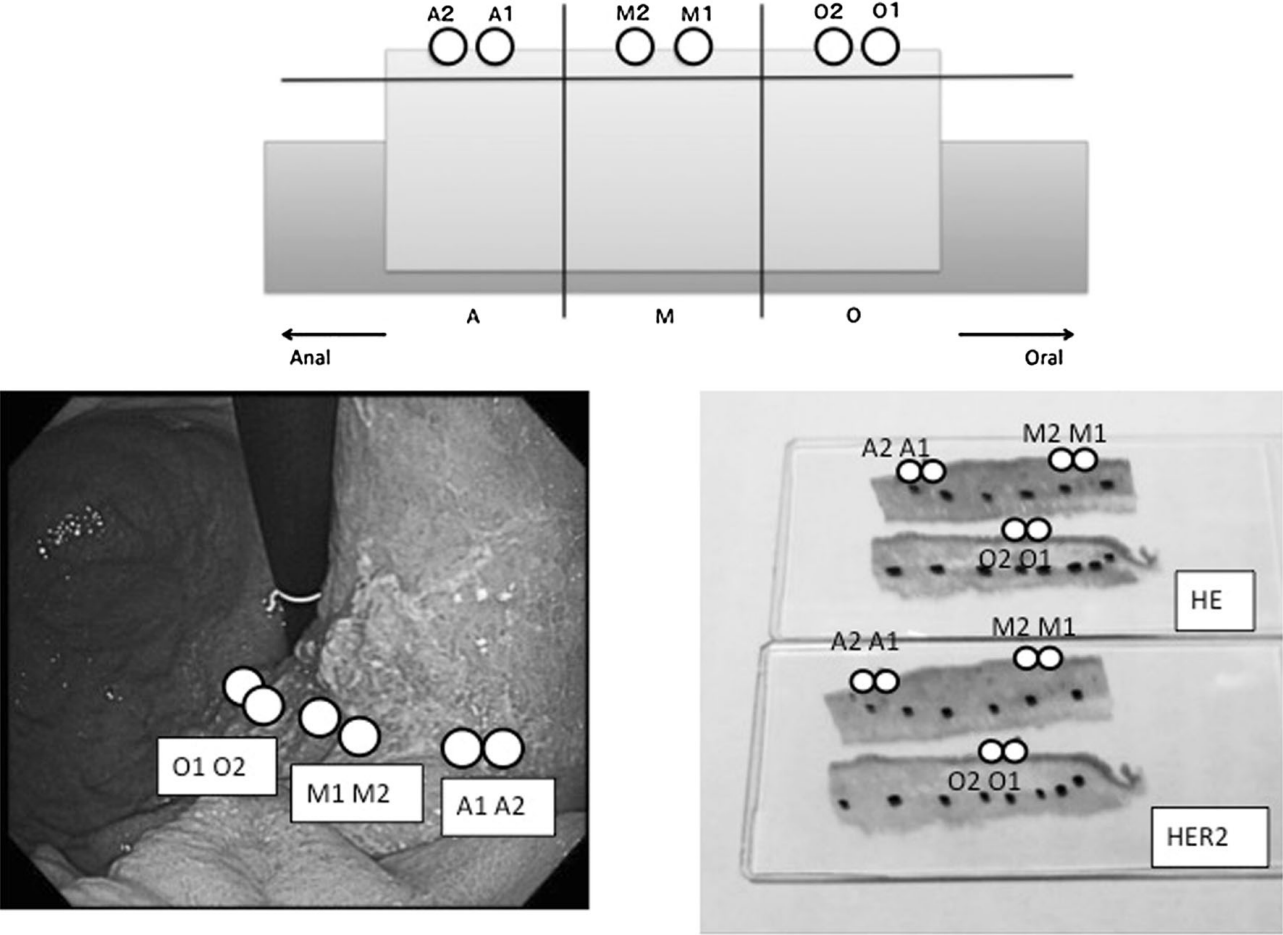
The “virtual biopsy.” We marked six positions on the prepared slide by a double-blind method: two were on the surface of the oral region (*O*), two were on the surface of the central region (*M*), and two were on the surface of the anal region (*A*). The *black dots* in the image at the *bottom right* indicate the tumor range (the tumor area was inside). The virtual biopsy sites are indicated by *white circles*. *HE* hematoxylin and eosin, *HER2* human epidermal growth factor receptor 2

To assess the concordance of the HER2 status and the histological subtype between biopsy specimens and the resection specimen, we collected data from six circles measuring 2 mm in diameter (“virtual” biopsy specimens) from the luminal layer of the hematoxylin and eosin stained slide and from the matched HER2-stained slide. Two virtual biopsy specimens were taken from the oral region, two were taken from the central region, and two were taken from the anal region from each tumor; see Fig. 2. The percentage of HER2-positive tumor cells in the virtual biopsy specimens was determined by assessing all tumor cells present in the biopsy specimen.

### Statistical analyses

A tumor was defined as being “heterogeneous” with respect to the histological subtype if two or more tumor regions showed a different histological subtype, and as being heterogeneous for HER2 expression if two or more tumor regions had a significantly different percentage of HER2-positive tumor cells.

To calculate the concordance rate between the biopsy specimens and the whole tumor, the percentage of HER2-positive cells in the biopsy specimens was cumulatively compared with the percentage of HER2-positive cells in the whole tumor. Thus, the percentage of HER2-positive cells in the first biopsy specimen was compared with that in the whole tumor, and then the percentage of HER2-positive cells in the first and second biopsy specimens, the percentage of HER2-positive cells in the first, second, and third biopsy specimens, etc. We also assessed whether the concordance rate between the biopsy result and whole tumor result was dependent on the region from which the biopsy specimen was taken.

**Fig. 3:**
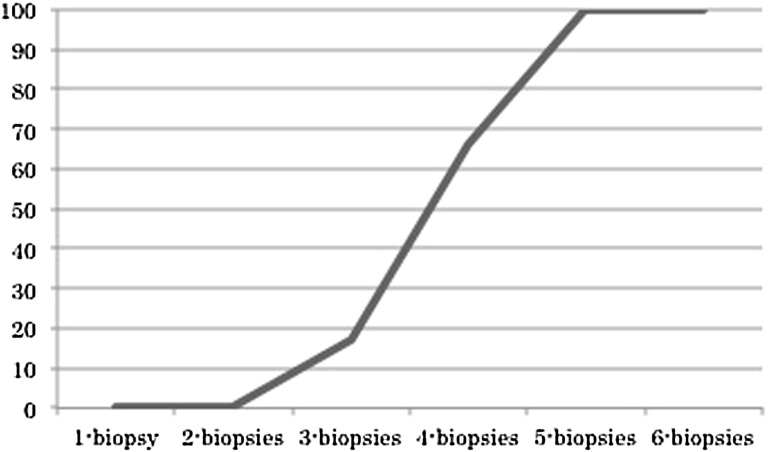
Human epidermal growth factor receptor 2 expression ratio related to the number of biopsy specimens

Using the HER2 expression status of individual virtual biopsy specimens, we analyzed the number of biopsy specimens required to diagnose “HER2 positivity.” The “HER2 expression ratio” (see Fig. 3) was calculated using probability statistics by calculating the number of combinations of *n* objects taken *r* at a time: *C*(*n*,*r*) = *n*!/[*r*!(*n* − *r*)!]. In this formula, the shorthand notation of *n*! (i.e., *n* factorial) is used. The denominator of the HER2 expression ratio was *C*(6,*r*′), and the numerator of the HER2 expression ratio was *C*(*n*′,*r*′). *n*′ is the number of HER2-positive virtual biopsy specimens in the case, and *r*′ is the number of biopsy specimens displayed on the *x*-axis of Fig. 3.

The *χ*^2^ test, the Mann–Whitney *U* test, and Holm’s test were used for categorical variables, continuous variables, and multiple comparisons, respectively. A *p* value of less than 0.05 was considered significant. Statistical analyses were performed using R (version 2.12.2).

## Results

### Patient characteristics

The patient and tumor characteristics of the study population are shown in Table 1. The median age was 71 years (range 53–84 years). The median tumor length from anal to oral was 44 mm (range 15–120 mm), from anterior to posterior was 30 mm (range 11–75 mm), and from luminal to deepest tumor infiltration in the wall was 8 mm (range 2–23 mm). The tumor was ulcerated in 16 patients (67 %).

**Table 1:** Patient and tumor characteristics

Tumor location	Number of patients	Percentage of patients
Upper third	7	29.2
Middle third	9	37.5
Lower third	8	33.3
Depth of invasion (pT)		
T1	13	54.2
T2	3	12.5
T3	3	12.5
T4	5	20.8
Lymph node status (pN)		
N0	10	41.7
N1	5	20.8
N2	5	20.8
N3	4	16.7
Ulceration		
With	16	66.7
Without	8	33.3
TNM stage		
I	12	50
II	6	25
III	5	20.8
IV	1	4.2
Heterogeneity of histological subtype		
Homogeneous	7	29.2
Intestinal type	6	25
Diffuse type	1	4.2
Heterogeneous	17	70.8
Luminal layer		
Homogeneous	9	37.5
Intestinal type	8	33.3
Diffuse type	1	4.2
Heterogeneous	15	62.5
Deeper layer		
Homogeneous	10	41.7
Intestinal type	6	25
Diffuse type	4	16.7
Heterogeneous	14	58.3

### Heterogeneity of histological subtype

Seven GCs (29 %) showed an identical histological subtype in all regions and all biopsy specimens and were therefore classified as homogenous. Six of these were classified as intestinal-type GC and one was classified as diffuse-type GC. When histological subtype heterogeneity was assessed by comparing layers (luminal vs deep), nine GCs (38 %) were classified as homogenous in the luminal layer, eight of which were intestinal-type GC and one of which was diffuse-type GC. Ten GCs (42 %) were classified as homogenous in the deeper layer, six of which were intestinal-type GC and four of which were diffuse-type GC. This suggests that the observed heterogeneity of the histological subtype may be related to the depth of invasion.

### Concordance of histological subtype between biopsy specimens and the resection specimen

Two biopsy specimens were taken from each luminal region—that is, six biopsy specimens per case. The results are shown in Table 2. In total, 19 biopsy specimens (13 %) were uninformative as they did not contain any tumor cells (eight, five, and six specimens from oral, central, and anal regions, respectively). In 23 GCs (96 %), the two biopsy specimens taken from the same region showed the same histological subtype, and in eight GCs (33 %), all six biopsy specimens showed the same histological subtype.

**Table 2:** The results of virtual biopsies

Quality of biopsy specimens	Number of patients	Percentage of patients
Informative biopsy specimens		
Oral	40	83.3
Central	43	89.6
Anal	42	87.5
Uninformative biopsy specimens		
Oral	8	16.7
Central	5	10.4
Anal	6	12.5
Concordance of histological subtype between biopsy specimen and region		
Same	23	95.8
Different	1	4.2
Concordance of histological subtype between six biopsy specimens		
Same	8	33.3
Different	16	66.6
Histological subtype by both biopsy specimens and region		
Oral		
Intestinal type	13	54.2
Diffuse type	1	4.2
Central		
Intestinal type	10	41.7
Diffuse type	5	20.8
Anal		
Intestinal type	11	45.8
Diffuse type	1	4.2

In the luminal oral region, 13 GCs (54 %) were classified as intestinal-type GC and one GC (4 %) was classified as diffuse-type GC by both biopsy specimens and region (concordance rate 58 %). In the luminal central region, ten GCs (42 %) were classified as intestinal-type GC and five GCs (21 %) were classified as diffuse-type GC by both biopsy specimens and region (concordance rate 63 %). In the luminal anal region, 11 GCs (46 %) were classified as intestinal-type GC and one GC (4 %) was classified as diffuse-type GC by both biopsy specimens and region (concordance rate 50 %).

### Heterogeneity of HER2 expression

The median percentage of HER2-positive tumor cells was 41.1 % (range 2.0–74.2 %), 34.0 % (range 0.0–90.7 %), 30.8 % (range 0.0–99.7 %), and 13.5 % (range 0.0–98.0 %) per case, layer, region, and biopsy specimen, respectively. Twenty-three GCs (96 %) had HER2-positive tumor cells in all luminal regions. The remaining one had HER2-positive tumor cells in the two lateral regions and had no HER2-positive tumor cells in the central region because of the presence of an ulceration. Five GCs (21 %) had no HER2-positive tumor cells in the deeper regions. The percentage of HER2-positive tumor cells was significantly different among regions in all GCs, and therefore all GCs were classified as heterogeneous for HER2 expression.

The percentage of HER2-positive tumor cells was significantly higher in the luminal layer than in the deeper layer [median percentage of HER2-positive tumor cells in the luminal layer of 60.3 % (range 3.9–90.7 %) vs 21.7 % (range 0.0–67.2 %) for the deeper layer; *p* < 0.001]. For the three different regions within the luminal layer, the percentage of HER2-positive tumor cells was higher in the lateral (anal and oral) regions than in the central region, although this did not reach statistical significance [median percentage of HER2-positive cells in the luminal anal region of 58.3 % (range 4.3–92.2 %) vs 65.0 % (range 4.2–99.7 %) for the luminal oral region and 57.2 % (range 0.0–95.0 %) for the luminal central region; *p* = 0.98].

The median percentage of HER2-positive tumor cells in the virtual biopsy specimens was highest in the oral region and lowest in the anal region [median percentage of HER2-positive cells in the oral region was 34.5 % (range 0–98 %) vs 11.5 % (range 0–91 %) in the central region and 0 % (range 0–98 %) in the anal region; *p* = 0.027 (oral vs central) and *p* = 0.020 (oral vs anal)].

### Concordance of HER2 expression between biopsy specimens and the resection specimen

Two biopsy specimens were taken from each luminal region—that is, six biopsy specimens per case. For the HER2-stained slides, 19 biopsy specimens (13 %) were uninformative as they did not contain any tumor cells (eight, five, and six specimens from oral, central, and anal regions, respectively). In 12 GCs (50 %), the two biopsy specimens taken from the same region showed a difference in the percentage of HER2-positive cells of over 87.2 %. Only two GCs (8 %) showed no significant difference in the percentage of HER2-positive tumor cells in all six biopsy specimens.

The concordance of the percentage of HER2-positive tumor cells between biopsy specimens and the resection specimen is shown in Table 3. In 17 GCs (71 %), 16 GCs (67 %), and 13 GCs (54 %), the percentage of HER2-positive cells in either one or two biopsy specimens from the luminal oral, central, or anal region, respectively, was not significantly different from the percentage of HER2-positive cells from the whole region of origin. In 12 GCs (50 %), 9 GCs (38 %), and 6 GCs (25 %), the percentage of HER2-positive cells in both biopsy specimens from the luminal oral, central, or anal region, respectively, was not significantly different from the percentage of HER2-positive cells in the region of origin. In 19 GCs (79 %), 19 GCs (79 %), and 15 GCs (63 %), the percentage of HER2-positive cells in either one or two biopsy specimens from the luminal oral, central, or anal region, respectively, was not significantly different from the percentage of HER2-positive cells in the whole tumor. In 12 GCs (50 %), 10 GCs (42 %), and 6 GCs (25 %), the percentage of HER2-positive cells in the both biopsy specimens from the luminal oral, central, or anal region, respectively, was not significantly different from the percentage of HER2-positive cells in the whole tumor. The concordance rate between the percentage of HER2-positive cells in the biopsy specimens and the percentage of HER2-positive cells in the whole tumor was highest (79 %) when biopsy specimens were obtained from the oral region and was lowest (63 %) when the anal region was sampled.

**Table 3:** The concordance of the percentage of human epidermal growth factor receptor 2 expression between biopsy specimens and the resection specimen

	Number of patients	Percentage of patients
One or two biopsy specimens from the region versus the whole region		
Oral	17	70.8
Central	16	66.7
Anal	13	54.2
Both biopsy specimens from the region versus the whole region		
Oral	12	50.0
Central	9	37.5
Anal	6	25.0
One or two biopsy specimens from the region versus the whole tumor		
Oral	19	79.2
Central	19	79.2
Anal	15	62.5
Both biopsy specimens from the region versus the whole tumor		
Oral	12	50.0
Central	10	41.7
Anal	6	25.0

### Relationship between histological subtype and HER2 expression per region

On the basis of the resection specimen, 22 HER2-positive GCs (92 %) were classified as intestinal-type GC and two (8 %) were classified as diffuse-type GC (*p* < 0.001)

### HER2 expression per tumor region

**Fig. 4:**
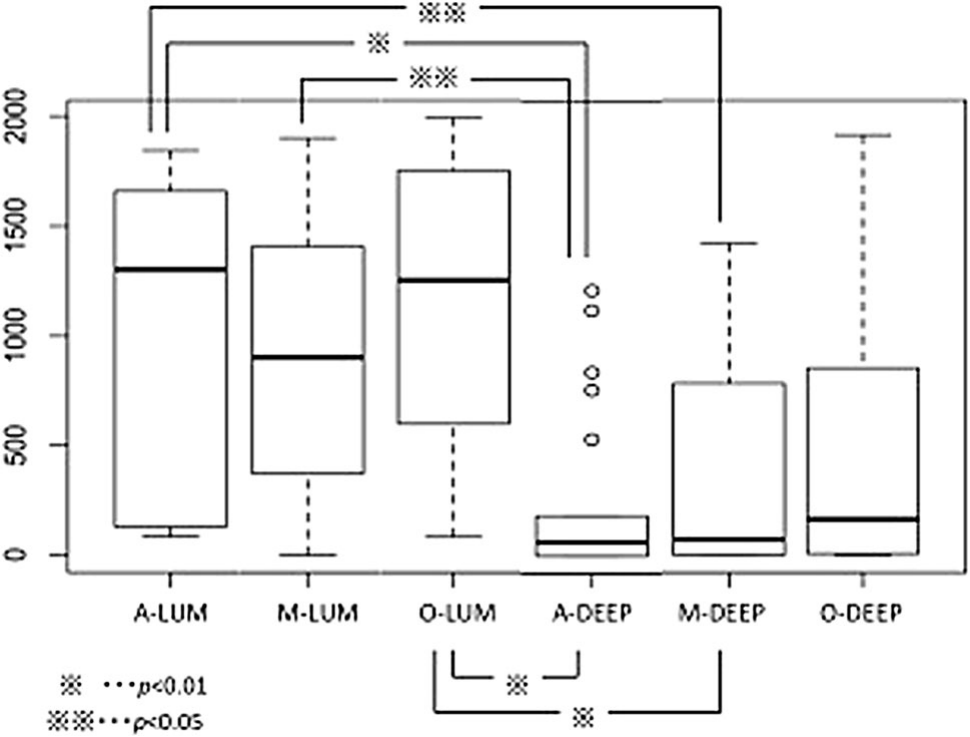
The result of Holm’s test for cells expressing human epidermal growth factor receptor 2 which were counted individually. *A* anal region, *DEEP* deeper layer, *LUM* luminal layer, *M* central region, *O* oral region

HER2-positive cells were counted for all regions separately and compared between regions. Luminal regions had significantly more HER2-positive cells than deeper regions (*p* < 0.001; Fig. 4), and lateral parts of the tumor (round wall side “O” and “A”) tended to have more HER2-positive cells than central regions.

### Estimation of the number of biopsy specimens needed for reliable HER2 testing based on “virtual biopsy” specimens

The proportion of HER2-expressing GCs detected by using different numbers of biopsy specimens is shown in Fig. 3. Although the ideal number of biopsy specimens was more than six, HER2 expression was detected in all GCs for five or more biopsy specimens.

## Discussion

The recent ToGA trial showed that the addition of trastuzumab therapy to chemotherapy significantly improves survival of patients with HER2-expressing advanced or metastatic GC, underscoring the importance to accurately identify patients eligible for this treatment [3]. However, reported rates of HER2 positivity differ considerably in the current GC literature [19, 20].

In this pilot study, we used material from previously classified HER2-positive GC resection specimens to quantitatively and qualitatively assess the heterogeneity of the histological subtype and HER2 expression in different regions of the tumor to identify the minimum number of biopsy specimens needed to achieve the highest concordance between biopsy specimen and resection specimen HER2 status. HER2-expressing tumor cells were preferentially located in the luminal lateral layer of the tumor, which coincides with the location of the well-differentiated tumor cells. Poorer differentiation in deeper layers has been described previously in GC [21]. It has been described previously that ulcerated central tumor areas were usually HER2 negative and should be avoided when sampling tumors endoscopically. Our virtual biopsy study indicates that five tumor-containing biopsy specimens are sufficient to have the same HER2 expression status as in the resection specimen. This supports the recommendation of the National Comprehensive Cancer Network guidelines for more than six biopsy specimens in order to diagnose HER2 expression in GC.

HER2 expression was detected in all cases by taking five biopsy specimens from the tumor, and the oral region seems to be the “optimal” location for sampling to determine the HER2 status in GC with a high level of confidence.

In conclusion, this is the first study that has demonstrated and quantified spatial heterogeneity of HER2 expression in GC and showed preferential expression of HER2 in the luminal and lateral parts of the tumor, supporting the validity of using endoscopic biopsy samples for HER2 testing. The preferential location of HER2-positive tumor cells coincided with the preferential location of well-differentiated tumor cells in GC. The achievable high concordance between HER2 biopsy specimen and resection specimen status for a minimum of five tumor-containing biopsy specimens is encouraging. We are aware that this is a retrospective study in a relatively small number of specimens, and thus a larger prospective study is required to confirm the findings from our pilot study before guidelines can be issued on endoscopic sampling of GCs for HER2 testing.
